# The effectiveness of online team-based learning in introduction to medical ethics education for medical students at a medical college of Nepal: a pilot study

**DOI:** 10.1186/s12909-022-03813-w

**Published:** 2022-11-08

**Authors:** Nuwadatta Subedi, Neelu Hirachan, Sabita Paudel, Bijayata Shrestha, Anju Pradhan, Anish Subedee, Xiaodan Li

**Affiliations:** 1 Department of Forensic Medicine, Gandaki Medical College Teaching Hospital and Research Center, Gandaki Province, Pokhara, Nepal; 2 Department of Pharmacology, Gandaki Medical College Teaching Hospital and Research Center, Gandaki Province, Pokhara, Nepal; 3 Department of Oral Pathology, Gandaki Medical College Teaching Hospital and Research Center, Gandaki Province, Pokhara, Nepal; 4 Department of Pathology, BP Koirala Institute of Health Sciences, Dharan, Nepal; 5 Department of Radiology, Narayani Samudayik Hospital, Bharatpur, Nepal; 6 Department of Internal Medicine, Zhujiang Hospital, Director of Teaching and Research, Southern Medical University, Guangzhou, China

**Keywords:** Active learning, Code of ethics, Medical education, Medical ethics, Collaborative learning

## Abstract

**Background:**

The effectiveness of online classes is always a concern, and it can be overcome by opting for active learning strategies like team-based learning (TBL). This study was conducted to find out the effectiveness of online TBL as an active learning strategy. We also aimed to explore the satisfaction and perception of students toward TBL.

**Methods:**

This is a mixed-method study conducted among 29 third-year Bachelor of Medicine and Bachelor of Surgery (MBBS) students of Gandaki Medical College using purposive sampling method in the duration of January to September 2021. Three two hours online TBL sessions were used for teaching introduction to medical ethics. The individual readiness assurance test (IRAT) scores were compared to the group readiness assurance test (GRAT) scores to evaluate the effect of TBL through cooperative learning. Learner reactions and satisfaction of students towards TBL were assessed using a validated questionnaire comprising of a five-point Likert scale. An open-ended question asking the participants to describe their overall experience of the TBL sessions was also included to explore their perceptions towards TBL. The data were collected using Google form and exported to Microsoft Excel and the quantitative data were then analyzed using Statistical Package for Social Sciences (SPSS) version 16.0. To check the normal distribution of the data, Kolmogorov Smirnov and Shapiro-Wilk test were used. Non-parametric tests were used for the non-normally distributed data. P value of < 0.05 was regarded as significant. Thematic analysis was conducted for the qualitative data.

**Results:**

The median GRAT scores were significantly higher (p = 0.006 in TBL 1 and 0.001 in TBL 2) than IRAT scores. Learner reactions toward TBL sessions were positive as shown by the mean scores which were in the range of 3.59 to 4.66. Five themes were generated from the codes: “effective learning method”, “positive experience”, “gained knowledge”, “expression of gratitude” and “the way of conduction of the sessions”.

**Conclusion:**

Online TBL in medical ethics was effective as a teaching learning tool in our setting. The students were satisfied with the learning process and rated the learning strategy positively.

**Supplementary Information:**

The online version contains supplementary material available at 10.1186/s12909-022-03813-w.

## Background

Medical ethics is an important part of medical education, to empower physicians to make ethical decisions [1]. The importance of medical ethics and principles of professional practice has been realized globally and has been recommended by the World Federation of Medical Education to incorporate into the medical curriculum [2].

Medical students in different settings have reported frequent ethical conflicts during their training. The reason behind this has been linked to inadequate training in medical ethics during their foundation course [3]. Though the Nepal Medical Council has published the code of ethics which the medical practitioners have to abide by during their practice [4], they are not satisfactorily aware about most of its contents [5]. This could be the reason for the increased number of allegations related to medical negligence [6]. This highlights prioritizing medical ethics teaching by consolidating our existing university curriculum [6]. Along with this, it can also be facilitated by the use of active learning strategies like team-based learning (TBL) which can be used in a large number of students by dividing them into teams [7], and as such, this can be suitable in resource-limited settings as well.

TBL is becoming popular in higher education health disciplines as it facilitates health professional educators to provide students with the cost-effective and reliable experience to work in small teams to solve clinical problems. In TBL, the students have to practice and learn actively before the class, and this is the reason why it facilitates active learning. The students are motivated towards self-directed learning and they interact with their peers with active engagement which facilitates better teamwork practice all leading to enhanced academic results, particularly for the weaker students [8]. In a medical college of Nepal, TBL was presented to be an effective tool to teach Physiology classes and was also rated better than traditional lecture-based delivery by the students [9].

The onset the of COVID-19 pandemic has shifted the mode of delivery of education to online in most settings [10]. If the online classes are taken conventionally, particularly emphasizing the use of lecture-based delivery, its effectiveness can be compromised[11]. Amongst different teaching-learning strategies that can be used for active participation of students in online delivery, TBL can be one of them. Online TBL has also been successfully conducted to suffice as an interactive methodology during the pandemic [12]. The judicious use of video conferencing can optimize the TBL process and even reproduce face-to-face conditions. The use of online TBL has also been appreciated by majority of the students and responded to having developed better communication skills with their peers [13].

Although TBL has been proven to be an effective tool and online TBL has been implemented in other contexts [14], this has not been routinely used in Nepalese universities. The research questions of the study were: (1) What is the effectiveness of online TBL as a new tool for teaching introduction to medical ethics to MBBS students? (2) What is the perception of students towards online TBL? (3) Are the students satisfied with online TBL?

## Methods

This is a mixed methods study conducted among 29 third-year Bachelor of Medicine and Bachelor of Surgery (MBBS) students of Gandaki Medical College in the duration of January to September 2021. The portion of the study to find out the effectiveness of TBL by assessing the scores of TBL classes was pre-experimental with one-group pretest-posttest design. The qualitative portion of the study was by effected incorporating an open question “Describe your overall experience of the TBL sessions” to collect their perceptions of TBL.

The college is affiliated with Tribhuwan University and runs various undergraduate and postgraduate medical, dental, nursing, and paramedical courses. The MBBS course follows the curriculum prepared by the university and each batch consists of 100 students. The course is of 4.5 years duration followed by one year of compulsory internship. Medical ethics is included in the forensic medicine curriculum of the third year and teaching is normally done by lecture-based delivery and is usually covered in four to six hours. Though TBL was planned to be conducted among all the students of MBBS third year in July-August, the academic calendar was affected by the COVID-19 pandemic and most of them had upcoming supplementary examinations on short notice. The 35 students not having the examinations were informed of their voluntary participation in the TBL session in medical ethics through notifications by a Viber group. The students were given information about the method, schedule, time, facilitator, tutors, and the mode of delivery of the TBL session in a flyer prepared in pdf format with a link to Google form for registration. The flyer also contained information about the usefulness of medical ethics and the importance of learning through TBL. If they would be consenting to participate, they were told to register by filling out the form which included their name, roll number, email address, and phone number used for Viber. A total of 29 students consented to participate and registered for the event and all were enrolled in the study using the purposive sampling method.

Inclusion criteria consisted of the students of third-year MBBS consenting to participate in the study, whereas the students who do not have to get enrolled in a regular course of forensic medicine including medical ethics were excluded from the study.

## Data collection Method/s /Technique/s

A list of the students registering to participate was prepared and six groups were prepared manually at the discretion of the facilitator to generate four to five students in each group. The teams were prepared such that there was as much diversity in students’ characteristics as possible in terms of academic performance in the preceding year, gender balance, geographic origin, etc. The students were not allowed to select their groups and were informed of the selection process transparently [15]. They were informed that the group would be the same throughout the TBL sessions. The students were also asked to choose a team leader, a timekeeper, a presenter, and a recorder in each group voluntarily by themselves. TBL sessions were conducted in Zoom as an online platform as it was also used to conduct online classes earlier, and the students were accustomed to it. The students were further oriented about the use of the software, particularly for the use of breakout rooms. A predefined breakout room was created based on the groups of students. One lead facilitator oriented the three other tutors who were given the responsibility to monitor and facilitate two groups each. The students were asked to rename themselves starting with their group number in front of their names while participating in the class so that they can be assigned to the respective breakout rooms with ease. We followed the tips for conducting online TBL as suggested by Malik et al. [13].

The introduction to medical ethics education was covered using TBL consisting of three sessions, the first session was one hour and the two subsequent sessions were two hours each. The contents of the course included principles of medical ethics, ethical dilemma, informed consent, and the code of ethics for the medical professions published by the Nepal Medical Council. The first session was used to describe TBL and give out the instructional materials and create teams. The presentation and recording of the orientation session were made available to the students so that those who had missed the session could also be prepared and could join the remaining sessions. The remaining two sessions, TBL 1 and TBL 2 respectively were used to teach medical ethics using TBL. The course contents focused on the principles of medical ethics (autonomy, non-maleficence, beneficence, and justice), in TBL 1 and consent in medical practice and the code of ethics of Nepal Medical Council in TBL 2.

## Design of the online TBL in medical ethics education

**Fig. 1:**
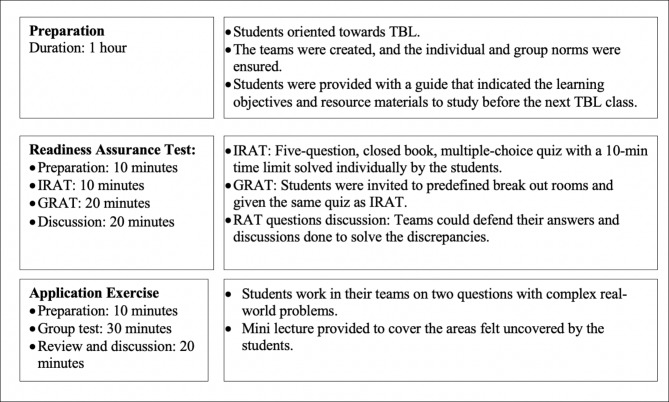
Design of TBL course. We had conducted two readiness assurance tests and application exercises each after one preparation class. TBL: Team based learning, IRAT: Individual readiness assurance test, GRAT: Group readiness assurance test, RAT: readiness assurance test

The design of the online TBL course is summarised in Fig. 1.

### Preparation

Three days before the class, all registered participating students were given a student guide that indicated the learning objectives and resource materials, and they were requested to study the provided materials individually before the class.

### Readiness assurance

The RAT questions were prepared in Google form. The questions on the Individual Readiness Assurance Test (IRAT) assess whether students understand and can apply important concepts of the medical ethics basic to the practice of medicine (e.g., consent and informed consent to treatment, physician-patient relationship, etc.) The answers in the Google form were downloaded to an excel sheet and graded later.

Immediately after IRAT, the preassigned teams of students were invited to predefined break-out rooms for Group Readiness Assurance Test (GRAT) and given the same quiz as IRAT in another Google form, where the students could discuss the answers online in the rooms and form a consensus for each answer. The group leader facilitated the discussion, and it was observed by one of the tutors who periodically switched on to each of the two preassigned groups. The facilitator also observed the team discussions randomly at any time, and it was pre-informed to all the participants. The recorder noted the team discussion in a notebook and reported to the team leader while the timekeeper observed the time and informed the group of the remaining time for discussion and making consensus answers. The team leader of each group was asked to fill out the Google form prepared separately for recording the GRAT and evaluated later. After the preassigned time, all the participants were taken to the main group and the team leader of each group was asked to write the answer number of each question simultaneously in the chat box after the lead facilitator asked to do so. The answers from each team were recorded by the instructor and the correct answer was revealed to them. If the teams did not agree to the answers, the discrepancies were addressed by asking the teams to defend their answers (RAT question discussion). This discussion phase was scheduled for 20 min to complete the first hour of class.

### Application exercise

When the students were known to have received the core concepts of the topic well after the RAT, the students were then taken forward to the application exercise. During the application exercise, the students worked in a team to solve two questions that required the ability to solve complex real-world problems. We had given 30 min to the teams to complete the exercise and 20 min to review.

In order to assess the learner reactions and satisfaction of students towards TBL, a validated questionnaire comprising of five-point Likert scale was used wherein students were requested to rank their responses: (1 = strongly disagree, 2 = disagree, 3 = somewhat agree, 4 = agree, and 5 = strongly agree) was used. It was based on that prepared by Ozgonul and Alimoghu after taking written permission from the author [16]. The form was modified to adjust the questions for online TBL, and the questionnaire was divided into five themes; “Organization, infrastructure, and resources”, “preparation and readiness”, “discussion”, “teacher” and “general”. It had undergone expert validation for the content and discrepancies were overcome. The IRAT scores were compared to the GRAT scores to evaluate the effect of TBL through cooperative learning. IRAT scores reflect students’ comprehension of content within the assigned reading before participating in learning in the team.

### Data management and analysis tools

The responses and the scores collected in online form were exported to Microsoft Excel and were then analyzed using Statistical Package for Social Sciences (SPSS) software version 16.0 (SPSS Inc., Chicago, IL, USA). To check whether the data were normally distributed, Kolmogorov Smirnov and Shapiro-Wilk tests were used. Non-parametric tests were used for the non-normally distributed data. The reliability of the tool was analyzed using Cronbach’s alpha. P-value of < 0.05 was regarded as significant.

The qualitative analysis of the responses obtained from the open question was done independently by a researcher who was not involved in quantitative analysis. Thematic analysis was used for qualitative analysis. After familiarization, coding of the responses was done. The codes were organized manually to look for patterns and themes were generated using an inductive approach. The themes were reviewed and redefined whenever necessary in order to better represent the data.

## Ethical consideration

Informed consent was obtained from the study participants before they were enrolled in the study, and we followed the Declaration of Helsinki during the research. Ethical approval was obtained from the Institutional Review Committee of Gandaki Medical College before commencing the study. (Ref no 119/077/78, dated 4th February 2021)

## Results

Among a total of 35 students invited for attending the TBL sessions, 29 participated and one student could not make up in the TBL 1.

The Cronbach’s Alpha value for the questionnaire of learner reactions and satisfaction towards TBL was 0.79, showing that the tool was reliable. The total scores of IRAT and GRAT in both the TBL were not normally distributed as analyzed by Kolmogorov Smirnov and Shapiro-Wilk test (p < 0.05). Wilcoxon Signed Ranks test was used to compare the IRAT and GRAT scores. The comparison of the scores of IRAT and GRAT is presented in Table 1. In both the TBL sessions, the median GRAT scores were significantly higher (p = 0.006 in TBL 1 and 0.001 in TBL 2) than IRAT scores. In TBL 1, there was an increase in score in GRAT as compared to IRAT of 16 students, the score was equal in nine participants while that of three students had decreased, while in TBL 2, the GRAT scores of 14 students increased as compared to IRAT while those of 15 remained the same.

All the students responded to the application exercise correctly in TBL 2 and by 64% in TBL 1. (Table 2) Learner reactions as satisfaction scores of the students on feedback form created for TBL sessions according to different themes are presented in Table 3. The responses were positive as shown by the mean scores as rated in the range of (1 = strongly disagree to 5 = strongly agree).

A total of 29 responses were obtained for the open question “Describe your overall experience of the TBL sessions”. Five themes were generated from the codes: “effective learning method”, “positive experience”, “gained knowledge”, “expression of gratitude” and “the way of conduction of the sessions”. The Length/brevity of the responses was taken as an indirect measure of the degree of involvement of the students.

Three frequently recurring themes were observed, viz. effective learning method, positive experience, and gained knowledge. The most commonly encountered narrative was ‘*fun and fruitful at the same time’.*

**Table 1:** Comparison of scores of readiness test in two TBL sessions

TBL Session	Readiness test	Median	Interquartile range	Z	P value
TBL 1 (n = 28)	IRAT	3	2–3	-2.731	0.006
	GRAT	4	3–4		
TBL 2 (n = 29)	IRAT	4	3.5-5	-3.416	0.001
	GRAT	5	5–5		

**Table 2:** Comparison of scores of application exercise in two TBL sessions

TBL Session	Correct answers of application exercise		
	**None** **n (%)**	**One** **n (%)**	**Both** **n (%)**
TBL 1 (n = 28)	5 (18)	5 (18)	18(64)
TBL 2 (n = 29)	0 (0)	0 (0)	29 (100)

**Table 3:** Learner reactions as satisfaction scores of the students on feedback form created for TBL sessions

Organization, infrastructure, and resources	Mean	Standard Deviation	95% CI
1. Information given at the start of the module about how TBL process runs was sufficient to understand the procedures well	4.34	0.60	4.13–4.56
2. Organization of the TBL session (duration, break time, exams, discussion process, etc.) was well	4.38	0.61	4.16–4.59
3. Online conditions in the learning environment were suitable	3.97	0.86	3.66–4.28
**Preparation and readiness**			
4. Self-study materials provided at the start of the module were comprehensive enough to gain required knowledge.	4.22	0.55	4.02–4.42
5. Individual/team test content was challenging enough to start discussion	3.59	0.87	3.28–3.91
**Discussion**			
6. Team assignments facilitated learning positively	4.62	0.49	4.45–4.80
7. Discussing all possible solutions facilitated the learning	4.66	0.48	4.48–4.83
8. This method helped us to show more systematic and logical approach to the patient	4.59	0.49	4.41–4.77
**Teacher**:			
9. The teacher helped us to better comprehend the subject by providing feedback, discussion, and explanations	4.66	0.48	4.48–4.83
10. The teacher supported our learning as much as he did in lectures	4.62	0.49	4.45–4.80
11. The teacher managed whole TBL process successfully	4.56	0.56	4.36–4.77
**General**:			
12. TBL increased my interest in medical ethics	4.59	0.49	4.41–4.77
13. I understood TBL classes better than other lectures	4.41	0.56	4.20–4.61
14. I focused on TBL sessions longer than other classes	4.53	0.57	4.33–4.74
15. I participated more actively in the TBL classes than other lectures	4.66	0.54	4.46–4.85
16. I think, the knowledge I gained in this TBL session will be more permanent than that I gained in lectures	4.41	0.61	4.18–4.63
17. Overall, I am satisfied with this TBL session	4.47	0.51	4.29–4.65

### Effective learning method

Almost half of the participants described TBL sessions as effective (words used: *effective, fruitful, beneficial*) and found the TBL method to be better than traditional learning methods. Some suggested it be used in future and in other fields also. Few of them even suggested for incorporation of TBL into the curriculum itself.

Better way of learning than regular formal classes

It was a great experience joining this TBL session - fruitful and fun at the same time. Looking forward to more sessions in upcoming days

very interactive ... it will be a great learning module if it is incorporated in our curriculum.

### Positive experience

Almost a third of the participants commented about their experiences and found their experiences to be enjoyable. The words used were *enjoyable, happy, fun*, and *glad*. An almost equal number of participants described the experience as *novel/new*. Some described the experience as interesting, and few described them as *long-lasting experiences.* None of the respondents had any negative experience, overall experience being positive.

### Gained knowledge

More than a third of the participants commented on the knowledge gained. Most used words like ‘*learned a lot, learned about doctor practices, learned something important, gained better knowledge’*. Four responses specifically mentioned learning about the ‘*medical ethics’* and ‘*doctor practices*.’

I did not know anything on the subject ... didn’t have interest in medical ethics. Now I can say there is massive positive change …gained lots of knowledge.

gained much concrete concept regarding this topic

### The way of conducting the sessions

Four responses mentioned the way sessions were conducted (words used: *amazingly conducted, engaging sessions*). Two persons mentioned that sessions were lengthy (*second session long, lengthy session*) and one person had an internet problem making online sessions difficult.

was quite skeptic earlier ... since this TBL was being conducted online, so many things could go wrong technically... but overall the session and the way teachers conducted the sessions was overwhelming

### Expression of gratitude

Eight responses expressed gratitude towards the teachers by thanking them for their effort as well as for providing the opportunity.

#### Length/brevity of the responses

Length of response can indirectly tell us about the degree of interest/involvement of the student. Almost two-thirds of the responses were of considerable length from which we can infer that the students must have had interesting and involving sessions. More than half responses had two to three sentences while only one response had a single word. Eight responses were quite long and elaborative, half of them having deeply personal accounts of the student’s experiences.

Being shy in public speaking, I never thought I would have such interacting session …the time I was assigned were really friendly with different perspective of thoughts... I am really happy and overwhelmed by this type of session...

## Discussion

We conducted this study with the objectives to find out the effectiveness of online TBL among the MBBS students and to explore the perception and satisfaction of the students towards it.

In our observation, the performance of students increased in teams as suggested by the significant increase of GRAT from the IRAT scores in both TBL sessions. Most of the students could solve the application exercise in the first TBL while all of them were successful to answer both of the application exercises in TBL 2. Though it could also be the effect of the difficulty level of exercise, it can also imply that the students could have developed better strategies to work in teams and solve the problems collaboratively in subsequent TBL sessions.

The online delivery of TBL is fundamentally the same as face-to-face TBL but some adaptations have to be made to facilitate the learning effectively. The major differences from face-to-face TBL are the physical separation of students from each other and the instructors, challenges in effective communication and engagement of students. There is also a necessity for clarification of queries to the questions by the instructors after each question separately rather than doing it at the end [13]. We had followed the mechanisms to conduct online TBL effectively and it worked well in our setting. The continuity in the internet facility was maintained by power backup and it was not an issue from the side of the instructors. The students were also pre-informed to make provisions for basic facilities including power back up and they were satisfied with the suitability of online conditions in the learning environment as suggested by their reactions as satisfaction scores.

As suggested by the recent evidence, it has been seen that TBL can be conducted successfully online[12, 17] as well as the students can find it enjoyable, interactive, and it facilitates collaborative learning [18]. In our observation also, the students were highly satisfied with the process of online TBL. In their responses, they mentioned that this method of learning encouraged them to have active participation in the class, provide longer attentiveness, better interaction with peers and instructors, increased interest in the topic and aid in long term memory of the subject matter.

As we had done in our study, Gaber et al. [12] successfully conducted online TBL using Zoom Breakout rooms and the students were satisfied with the method. Evidence from Oman has shown online TBL to be effective to improve students’ learning and it was also welcomed by most of the students [19]. In an observation by DeMasi et al. [20], there were no significant differences in scores of performances in online or physical TBL, though the perception of students was better for the physical TBL than the online. While online TBL can increase learning and help to foster teamwork, the students have perceived a tremendous increase in workload and require a significant amount of time [21]. Online TBL has also been accepted by the stakeholders and has been proven to be a feasible method of teaching learning [22].

The COVID 19 pandemic has changed the pattern of medical education worldwide. In order to cope with the pandemic, most academic medical centres have developed virtual methods of education as well as health care deliveries which is expected to impart some change in the existing system and maintain that even after the end of the pandemic [23]. Though the online delivery of medical education is a need of the hour, the effectiveness of learning should be considered and the strategies which can impart active learning should be used.

TBL is an effective form of learning, and the students have a positive perception towards it. It can also be effectively conducted online taking the mechanisms to adapt to the online environment into consideration. This will not only actively engage the students but also help them make them competent to handle real-world problems, work collectively, and facilitates long-term memory.

### Limitations and strengths of the study

Though the session was conducted following all the protocols of online TBL, the environment could have been better controlled if done physically. The online delivery could have threatened the validity of the pre-experimental study design. The sample size was also less. As the study was conducted during the second wave of the COVID-19 pandemic, and the students were used to the conventional online class, our results could have been biased. The use of online TBL probably for the first time in Nepal is the strength of our study. Though online classes have been criticized for not being interactive, online TBL could overcome the shortcoming as per our observation.

The study can be further extended to include more students and other topics too. We can strengthen the research design to experimental by including a control group or a comparison with other methods of intervention, such as didactic lectures. Further, longitudinal studies with analysis of the GPA scores can be conducted for generating reliable evidence for advocating its incorporation into the curriculum.

## Conclusion

Online TBL was effective as a teaching learning tool in our setting. The students were satisfied with the learning process and rated the learning strategy positively. The TBL sessions were a positive experience for students, sessions being successful in imparting the knowledge of the topic in an effective, interesting, and enjoyable way. Students found the sessions to be better than traditional lectures. Overall, the sessions were well conducted, and the students seemed to have had interesting and involving sessions. Online TBL can be more interactive and impart active learning to the students.

## Electronic supplementary material

Below is the link to the electronic supplementary material.

Supplementary Material 1

## Data Availability

The datasets used and/or analyzed during the current study are available from the corresponding author on reasonable request. The data are not available publicly due to privacy.
