# Supplementary material for: The effectiveness of online team-based learning in introduction to medical ethics education for medical students at a medical college of Nepal: a pilot study
Source: BMC Med Educ. 2022 Nov 8;22:766. doi: 10.1186/s12909-022-03813-w (PMC9643934; doi:10.1186/s12909-022-03813-w)
Supplement: Supplementary file 1 — Supplementary Material 1 [file 12909_2022_3813_MOESM1_ESM.docx]

**Survey Questionnaire for TBL sessions**

**Title:** The effectiveness of online team-based learning in introduction to medical ethics education for medical students at a medical college of Nepal: A pilot study

**Description:** Team-Based Learning (TBL) is a well-defined instructional strategy where small groups of students work together to solve problems in an instructor-led but learner-centered environment with well-structured processes of individual and group accountability. In TBL, there is brainstorming and recalling of prior theoretical knowledge.

The purpose of this project is to find out the effectiveness of online TBL and to explore perception and satisfaction of students towards the TBL sessions.

Be sure to read the answer options carefully. Please be honest and report your true reaction to each question by a tick for the response that best describes your answer.

**Organization, infrastructure, and resources**

| S.N | Statement | Strongly agree | Agree | Neutral | Disagree | Strongly disagree |
| --- | --- | --- | --- | --- | --- | --- |
| 1 | Information given at the start of the module about how TBL process runs was sufficient to understand the procedures well |  |  |  |  |  |
| 2 | Organization of the TBL session (duration, break time, exams, discussion process, etc.) was well |  |  |  |  |  |
| 3 | Physical conditions in the learning environment were suitable |  |  |  |  |  |

**Preparation and readiness**

| S.N | Statement | Strongly agree | Agree | Neutral | Disagree | Strongly disagree |
| --- | --- | --- | --- | --- | --- | --- |
| 4 | Self-study materials provided at the start of the module were comprehensive enough to gain required knowledge |  |  |  |  |  |
| 5 | Individual/team test content was challenging enough to start discussion |  |  |  |  |  |

Discussion

| S.N | Statement | Strongly agree | Agree | Neutral | Disagree | Strongly disagree |
| --- | --- | --- | --- | --- | --- | --- |
| 6 | Team assignments facilitated learning positively |  |  |  |  |  |
| 7 | Discussing all possible solutions facilitated the learning |  |  |  |  |  |
| 8 | This method helped us to show more systematic and logical approach to the patient |  |  |  |  |  |

Teacher

| S.N | Statement | Strongly agree | Agree | Neutral | Disagree | Strongly disagree |
| --- | --- | --- | --- | --- | --- | --- |
| 9 | The teacher helped us to better comprehend the subject by providing feedback, discussion, and  Explanations |  |  |  |  |  |
| 10 | The teacher supported our learning as much as he did in lectures |  |  |  |  |  |
| 11 | The teacher managed whole TBL process successfully stand the procedures well |  |  |  |  |  |

General

| S.N | Statement | Strongly agree | Agree | Neutral | Disagree | Strongly disagree |
| --- | --- | --- | --- | --- | --- | --- |
| 12 | TBL increased my interest in medical ethics |  |  |  |  |  |
| 13 | I understood TBL classes better than other lectures |  |  |  |  |  |
| 14 | I focused on TBL sessions longer than other classes  the procedures well |  |  |  |  |  |
| 15 | I participated more actively in the TBL classes than other lectures |  |  |  |  |  |
| 16 | I think, the knowledge I gained in this TBL session will be more permanent than that I gained in lectures |  |  |  |  |  |
| 17 | Overall, I am satisfied with this TBL session |  |  |  |  |  |

Open question.

18. Describe your overall experience of the TBL sessions.

**Scoring tool**

Roll Number:

Academic year:

| **TBL 1** | **Score obtained** | **Remarks** |
| --- | --- | --- |
| IRAT score |  |  |
| GRAT score |  |  |
| Application exercise |  |  |
|  | | |
| **TBL 2** |  |  |
| IRAT score |  |  |
| GRAT score |  |  |
| Application exercise |  |  |
